# Neglected cause of retinal detachment: a hospital-based case-control study on occupational heavy lifting as a risk factor

**DOI:** 10.1186/s42506-021-00091-0

**Published:** 2021-11-16

**Authors:** Nayera S. Mostafa, Radwa Nabil El Shereif, Ayat F. Manzour

**Affiliations:** 1 Department of Community, Environmental and Occupational Medicine, Faculty of Medicine, Ain Shams University, Ramsis Street, Abbaseya, Cairo, 11566 Egypt; 2 Department of Ophthalmology, Faculty of Medicine, Ain Shams University, Ramsis Street, Abbaseya, Cairo, 11566 Egypt

**Keywords:** Heavy lifting, Occupational lifting, Retinal detachment, Work factors, Work environment

## Abstract

**Background:**

Heavy lifting may lead to sudden increase in venous, intra-abdominal, and intraocular pressure which in turn may cause retinal detachment (RD). The epidemiological evidence for this association is still inconclusive. This study was carried out to investigate the relationship between occupational heavy lifting and RD.

**Methods:**

A case-control study was carried out on 151 RD cases and 113 controls free of RD attending the ophthalmology outpatient clinic at Ain Shams University. Personal, medical, and occupational data were collected using interview questionnaires in addition to conducting full ophthalmologic examination.

**Results:**

The mean age of study participants was 45.8 ± 9.1 years (46.8 ± 8.9, 44.4 ± 9.2 for RD cases and controls respectively). Statistically significant differences were found between cases and controls regarding years of working, occupational categories, frequency of occupational heavy lifting, non-work heavy lifting, history of head trauma, history of eye surgeries, and family history of RD. Multivariate logistic regression analysis showed that lifting (Odds ratio (OR) = 4.8, *p* < 0.0001), history of head trauma (OR = 3.3, *p* = 0.013), diabetes mellitus (DM) (OR = 4.96, *p* < 0.0001), and previous eye surgeries (OR = 3.5, *p* = 0.003) increased the risk of RD.

**Conclusion:**

Occupational heavy lifting is associated with RD. Occupational categories, duration of lifting heavy objects during work and family history of RD had a significant effect on RD. An ergonomic approach should be adopted and practiced as it has a significant impact on reducing the risks of lifting and carrying heavy objects. The workplace’s design (including having appropriate mechanical aids available) is also of significant importance to reducing the risks. During the patient’s visit, ophthalmologists should consider and look for the occupational heavy lifting history as a potential risk factor of the patient’s symptoms.

## Introduction

Retinal detachment (RD) is a serious ophthalmologic disorder, which can lead to irreversible loss of vision. It occurs when sub-retinal fluid accumulates in the space between the neurosensory retina and the underlying retinal epithelium. According to the mechanism of sub-retinal fluid accumulation, RD has been classified into rhegmatogenous, tractional, exudative, and combined tractional-rhegmatogenous types of RD. The most common type of RD is rhegmatogenous retinal detachment (RRD) where a tear in the retina occurs leading to fluid accumulation [1].

The Canadian Association of Optometrists [2] mentioned that the well-known causes of RD include head trauma, severe myopia, diabetes, and previous eye surgery. Previous studies have revealed an association between heavy lifting involving the valsalva manoeuvre (forceful exhalation against a closed glottis), and a sudden increase in venous, intra-abdominal, and intraocular pressure [3, 4]. This increased pressure causes a decompensation at the levels of the retinal capillary bed, resulting in retinal hemorrhages: either unilateral or bilateral [5]. Uncertainty persists about the effect of some types of exercises or activities on the intra-ocular pressure [6].

RD is a serious disease; its advanced stages may lead to blindness [7]. Studies carried out for identification of risk factors are important both for primary and secondary prevention. A Scottish study found that the incidence of RRD among people living in wealthier areas was twice that of poorer areas [8]. Likewise, an Italian population-based study indicated that the incidence of RRD incidents in manual workers was double the number of the incidents in non-manual workers [9].

Ireland is one of the countries which set ergonomic workplace laws and legislations. The Safety Health and Welfare at Work Regulations of 1993 included regulations specifically for lifting heavy objects. The Ergonomics Principle sets limits for the weight of any lifted load. Accordingly, a specific manual guide on weight limits was decreed for both males and females. Using mechanical aids and transport equipment in the workplace (such as trolleys and mobile raising platforms) will reduce manual handling of heavy weights [10].

Few studies were conducted on the relationship between heavy lifting and RD; however, we could not identify any such studies in Egypt. This study was carried out to investigate the relationship between occupational heavy lifting and RD.

## Methods

### Study design

#### A case-control study

The cases were patients suffering from RD while controls were patients seeking medical advice at the ophthalmology outpatient clinic and not suffering from RD.

### Study (target) population

Adult patients (18 years or older) seeking medical advice at Ain Shams University ophthalmology outpatient clinic.

#### Inclusion criteria

Cases are defined based on the following criteria:

1. Symptoms:

i. A sudden dramatic increase in the number of floaters.
ii. Very brief onset of photopsia (flashes of light) in the extreme peripheral part of  visual field.
iii. Adense shadow that starts in peripheral visual field and slowly progresses towards the central vision.
iv. An impression of veil or black curtain over the field of vision.

2-Signs:

i. Acute onset of deterioration in the visual acuity.
  - Fundus examination if the media is clear and shows rhegmatogenous or tractional RD.
  - B-scan US if the media is hazy (e.g.,: in case of cataract).

#### Exclusion criteria

i. Diabetic retinopathy.
ii. Ischemic retinopathy.
iii. Exudative RD (choroidal or inflammatory causes)

### Sampling and sample size

A sample of 106 participants per group was calculated using the odds ratio of index 1 = 3.57 [11] and prevalence of RD of 6% among controls with the alpha error = 0.05 and the power of study = 80%. The sample increased to include a total of 264 participants divided as 151 cases and 113 controls. A consecutive non-random sample of patients attending the clinic during 6 months according to the criteria of inclusion was enrolled in the study.

### Study setting and time

Data was collected at the Ain Shams University ophthalmology outpatient clinic in 2018. An interview questionnaire was filled by the researcher, in addition to a medical examination sheet filled by the ophthalmologist.

### Study tool

I- An interview questionnaire which included the following:

1. Participant’s personal information: age, gender, smoking status.
2. Participant’s occupational data (work nature, duration of working years, etc.). The occupations were classified according to history of occupational heavy lifting into 2 categories: a group including occupations strongly associated with heavy lifting (e.g., builders, construction, and storage workers) and another group including occupations not typically associated with heavy lifting and in which heavy lifting is unlikely to occur (e.g., technicians and office workers). Occupational heavy lifting is defined by the Bureau of Labor statistics as carrying about 5–11 kg constantly, 11.5–22.5 kg frequently or 23–45 kg occasionally at work [12].
3. Participant’s history of heavy lifting: frequency in relation to total working time, and average weight lifted at work.
4. Participant’s medical and ophthalmologic history in addition to other risk factors to confirm the diagnosis of RD and investigate the causal relationship with some factors.

II-An eye examination including the following:

  1-Assessment of visual acuity for near and distant objects (which was sometimes not possible to be done, so finger-counting tests and light-perception tests were done instead.

  2-Checking pupillary reaction: a dilated fixed pupil condition may be a result of trauma, Marcus-Gunn pupil can occur with any disturbance of afferent pupillo-motor pathway, including RD.

  3-Bilateral measurement of intraocular pressure: relative hypotony of RD eye ˃ 4–5 mmHg is common.

  4-Examination of the anterior segment of the eye using slit lamp: to detect signs of trauma, associated cataract, and uveitis.

  5**-**Examination of the vitreous body for signs of pigments or tobacco dust: which is suggestive of retinal tear.

  6-Dilated fundus examination using indirect ophthalmoscopy: to indicate the type of RD, presence of retinal tear or hole and its site and size, macular state, blood vessels, presence or absence of hemorrhages, and optic disc state.

Three types of RD could be detected by examination:

  1-Rhegmatogenous RD:

This results when a tear or break occurs in the neurosensory retinal layer and allows liquefied vitreous to seep between and separate the sensory and the retinal pigment epithelial layer; this is in addition to the presence of holes within an area of lattice degeneration.

  2-Tractional RD:

This results from adhesions between the vitreous gel and fibro-vascular proliferation and the retina, it occurs more commonly in diabetic and traumatic retinopathies. Fibrous or fibro-vascular tissues (epiretinal membranes) with areas of traction on retina are seen in fundus examinations.

3- Exudative RD:

This results from exudation of material into the sub-retinal spaces from retinal vessels (as in hypertension, central retinal vein occlusion, vasculitis, or papilledema).

### Statistical analysis

Data was coded, revised, and analyzed by the Statistical Package of Social Sciences (SPSS) version 20 [13]. Descriptive statistics was done using numbers and percentages for categorical variables, with the mean ± SD and range for quantitative variables. A Student’s *t* test was used to compare quantitative variables between study groups. A chi-square test was used to compare categorical variables. Multivariate logistic regression, including factors which were significant in the univariate analysis, was done. The statistically significant level was determined at *p* < 0.05.

## Results

The current study included 151 cases of RD and 113 patients free of RD seeking medical advice at an ophthalmology clinic. The mean age of study participants was 45.8 ± 9.1 years (46.8 ± 8.9, 44.4 ± 9.2 for RD cases and controls respectively). Table 1 shows that there was a statistically significant difference regarding the history of head trauma and the family history of RD (the proportion is higher among cases in both variables *p* < 0.0001). Regarding sex, the difference was also statistically significant (the proportion is significantly higher in male cases *p* = 0.021) while the difference was not significant regarding smoking status or age quartiles.

**Table 1:** Comparison between cases of retinal detachment and controls regarding socio-demographic characteristics and family history of retinal detachment, Ain Shams Outpatient Ophthalmology clinic, Cairo, Egypt, 2018

Variable	Control ***n*** (%)	RD case ***N*** (%)	Chi-square	***p*** value
**Age quartiles**^**§**^			4.968	0.174
**≤ 39 (Q1)**	39 (52.7)	35 (47.3)		
**39–47 (Q2)**	27 (43.5)	35 (56.5)		
**47–53 (Q3)**	26(37.7)	43 (62.3)		
**> 53 (Q4)**	21(35.6)	38(64.4)		
**Sex**			5.316	0.021*
**Male**	73(38.4)	117(61.6)		
**Female**	40(54.1)	34(45.9)		
**Smoking status**			.000	0.991
**Non-smoker**	83(42.8)	111(57.2)		
**Current smoker**	30(42.9)	40(57.1)		
**History of head trauma**			14.697	< 0.0001*
**Yes**	10(19.2)	42(80.8)		
**No**	103(48.6)	109(51.4)		
**Family history of RD**			23.024	< 0.0001*
**Yes**	7(13.5)	45(86.5)		
**No**	106(50.2)	105(49.8)		

* Significant

^§^ Age grouping was based on median and quartile ranges

Table 2 shows that there was a statistically significant difference regarding the number of working years (*p* < 0.0001). There were no significant differences regarding other occupational data (working days or hours, average weight lifted).

**Table 2:** Comparison between cases of retinal detachment and controls regarding occupational data

	Case Mean ± SD	Control Mean ± SD	***t*** test	***p*** value
**Working years**	21.23 ± 8.82	16.81 ± 8.52	3.814	< 0.0001*
**Working days/week**	6.46 ± 0.59	6.34 ± .51	1.648	0.101
**Working hours/day**	7.05 ± 1.59	7.04 ± .97	0.065	0.95
**Average weight lifted**	1.83 ± 1.18	1.89 ± 1.12	− 0.332	0.74

* Significant

Table 3 shows a statistically significant difference between cases and controls regarding the occupational categories, the frequency of occupational heavy lifting, and non-work heavy lifting (*p* = 0.012, *p* = 0.014, and *p* = 0.005 respectively).

**Table 3:** Comparison between cases of retinal detachment and controls regarding heavy lifting and history of head trauma, Ain Shams Outpatient Ophthalmology clinic, Cairo, Egypt, 2018

Variable	Control *n* (%)	Case *n* (%)	Chi-square	*p* value
**Occupational categories**				
**Associated with heavy lifting**	31(32.6)	64(67.4)	6.27	0.012*
**Unlikely associated with heavy lifting**	82(48.5)	87(51.5)		
**Frequency of occupational heavy lifting#**				
**Half or more of working time**	38(40.4)	56(59.6)	5.98	0.014*
**Less than half of working time**	32(61.5)	20(38.5)		
**Lifting heavy objects other than work**				
**No**	79(49.7)	80(50.3)	7.73	0.005*
**Yes**	34(32.4)	71(67.6)		

* Significant

# Among heavy lifters only

Table 4 shows statistically significant differences regarding eye surgery (< 0.0001). The complications category did not differ significantly between both groups.

**Table 4:** Comparison between cases of retinal detachment and controls regarding previous eye surgery and its complications, Ain Shams Outpatient Ophthalmology clinic, Cairo, Egypt, 2018

Variable	Control ***n*** (%)	Case ***n*** (%)	Chi-square	***p*** value
**Previous eye surgery**				
**No**	88(53)	78(47)	19.04	< 0.0001*
**Yes**	25(25.5)	73(74.5)		
**Complications of eye surgery**				
**No complications**	24(31.2)	53(68.8)	3.81^**#**^	0.061
**Vitreous loss**	1(6.7)	14(93.3)		

* Significant

# Fisher’s exact test

A significant difference was found between cases and controls regarding myopia (*p* = 0.05), cardiovascular (*p* = 0.021), and prostatic diseases (*p* = 0.046) as comorbidities, while non-significant differences are found regarding hypertension, respiratory diseases or constipation. Independent variables—which were significant in univariate analysis—were included in a binary logistic regression model as shown in Table 5. The following variables independently increased the risk of RD: occupational category is associated with heavy lifting (odds ratio (OR) = 4.8, *p* < 0.0001), history of head trauma (OR = 3.3, *p* = 0.013), DM (OR = 4.96, *p* < 0.0001), and undergoing previous eye surgeries (OR = 3.5, *p* = 0.003).

**Table 5:** Multivariate logistic regression of studied risk factors of RD

				95% C.I. for odds ratio	
Variable	***B***	***p*** value	Odds ratio (OR)	Lower	Upper
**Gender (female)**	1.07	0.064	2.905	0.94	8.99
**Occupational category (associated to heavy lifting)**	1.57	< 0.0001	4.82*	2.14	10.88
**Family history(positive)**	2.77	< 0.0001	15.91*	4.84	52.33
**Working years**	0.002	0.947	1.002	0.95	1.05
**Heavy lifting other than work**	0.49	0.193	1.633	0.78	3.42
**Head trauma history**	1.19	0.013	3.295*	1.29	8.43
**Diabetes mellitus**	1.6	< 0.0001	4.967*	2.05	12.01
**Previous eye surgery**	1.26	0.003	3.522*	1.54	8.06
**CVS disease**	1.15	0.058	3.168	0.96	10.45
**Prostate**	− 0.66	0.575	0.517	0.05	5.17
**Constant**	− 2.48	0.089	0.084		

* Significant     CVS: cardiovascular system

*OR* odds ratio

## Discussion

The mean age of RD cases in the current study is 46.8 ± 8.9 years, which is close to the mean age of RD cases in Southwest Ethiopia [14], Addis Ababa [15], Indonesia [16], and Scotland [17]; this mean age is slightly lower than the mean age of RD cases in Netherlands [18], and Japan [19].

The current study found that occupations associated with heavy lifting carried more risk to RD (*p* = 0.012). An Italian study indicated that RD was associated with manual workers as twice as they were with non-manual workers [9]. Other Scottish studies **[**8, 20] found an association with high educational and economic standards to RD which are usually uncommon among manual workers in their country. As high body mass index may have an additional effect on intra-ocular pressure upon performing valsalva manoeuvre [21, 22], it would be beneficial to investigate the participants’ weight in further studies.

Mattioli et al. investigated the relationship between lifting heavy loads as a type of physical exertion [21] in one study and in another study involving occupational tasks [22]. In both studies, Mattioli et al. confirmed a relationship between RD and lifting heavy objects.

Regarding heavy lifting as a risk factor of RD, it was previously explained as a reason for elevated choroidal pressure and vitreal traction due to valsalva manoeuvre which may lead to valsalva hemorrhagic retinopathy [21]. In addition, a case report was published in 2014, describing a healthy 27-year-old Turkish construction worker, who suffered from valsalva retinopathy after heavy cement bag lifting [22]. Long ago, it was proved that heavy lifting is accompanied by an increase in the intraocular pressure [4]. Farioli et al. indicated similar findings in their Swedish study (relative risk (RR) = 2.68) [23]. A population-based study in Denmark also found no association between occupational heavy lifting and RD [24]. In their study, Curti et al. reported their limitations and some unadjusted confounders. They declared that details about heavy lifting as well as data about myopia were not collected (which they considered a possible confounder).

The current study found a significant difference between cases and controls regarding cardiovascular diseases; however, another study carried out in Sweden found weak association between CVS disease like hypertension and RD [25]. Strong associations of RD with eye surgery, eye or head trauma, severe myopia (all are known risk factors), and heavy lifting were found by Mattioli et al. [21]. Another study estimated that the risk of RRD and posterior vitreous detachment were significantly higher among patients with myopia [19].

Although the current study did not prove a significant difference between cases and controls regarding average weight lifted, a statistically significant difference was found regarding the frequency of occupational heavy lifting (*p* = 0.014) as cases lifted heavy objects for longer periods than controls did (half or more of the working time).

Mattioli et al. [21] analyzed the relation between RD and heavy lifting. In their analysis, they combined frequency and weight of loads lifted. The average lifting performed in 1 week (kg × frequency) was significantly associated with RD in both univariate and multivariate analyses.

### Study limitations

The study was limited by being a hospital-based one due to the nature of case-control study. Further studies on a wider scale using community controls may be more informative. Although this study tried to investigate heavy lifting, the self-reporting measurements of heavy lifting by the participants may be considered a limitation of the study. Another limitation is the lack of details of anthropometric measures and visual acuity of patients.

## Conclusions

RD is a serious ophthalmologic disorder, which can lead to irreversible loss of vision. Family history of RD showed a significant effect on the occurrence of RD. Occupational categories and duration of lifting heavy objects during work also had a significant effect on RD. Non-work heavy lifting (which may cause an additive effect to occupational heavy lifting) significantly and independently affected RD incidence (based on the multivariate regression model). It is recommended to follow the international regulations, like that of the International Labour Organization recommendations for heavy lifting in different working environments. An ergonomic approach has a significant impact on reducing the risks of lifting and carrying heavy objects. A good workplace design (having the use of appropriate aids available) is of utmost importance. It is also recommended for heavy lifting workers to seek medical advice early and regularly to avoid the occurrence of RD. Ophthalmologists should consider the occupational heavy lifting history during visits of all patients.

## Data Availability

Available on reasonable request from the corresponding author.

## Abbreviations

RD: Retinal detachment
RRD: Rhegmatogenous retinal detachment
